# Trough anticoagulant levels of high-dose versus standard-dose intravenous enoxaparin in patients undergoing trans-radial coronary angiography alone

**DOI:** 10.1186/s12872-026-05618-x

**Published:** 2026-02-19

**Authors:** Lun Wang, Qian Chen, Liang Wang, Dingding Zhang, Jingyi Li, Ran Tian, Hao Qian, Xueqing Zhu, Lihong Xu, Xinglin Yang, Tengyue Zhang, Yifan Liu, Zhenyu Liu, Wei Wu

**Affiliations:** 1 Department of Cardiology, Peking Union Medical College Hospital, Chinese Academy of Medical Sciences and Peking Union Medical College, No.1 Shuai Fu Yuan, Dongcheng District, Beijing, 100730 China; 2 Department of Laboratory Medicine, Peking Union Medical College Hospital, Chinese Academy of Medical Sciences and Peking Union Medical College, No.1 Shuai Fu Yuan, Dongcheng District, Beijing, 100730 China; 3 Center for Prevention and Early Intervention, National Infrastructures for Translational Medicine, Institute of Clinical Medicine, Peking Union Medical College Hospital, Chinese Academy of Medical Sciences and Peking Union Medical College, No.1 Shuai Fu Yuan, Dongcheng District, Beijing, 100730 China; 4 Clinical Pharmacology Research Center, Peking Union Medical College Hospital, Chinese Academy of Medical Sciences and Peking Union Medical College, No.1 Shuai Fu Yuan, Dongcheng District, Beijing, 100730 China

**Keywords:** Anticoagulation, Enoxaparin, Percutaneous coronary intervention

## Abstract

**Background:**

It was presumed that enoxaparin 0.5 mg/kg, the guideline-recommended anticoagulant regimen for percutaneous coronary intervention (PCI), could achieve target anticoagulation for 90 min, which, however, was based on the results of pharmacokinetic simulation. This study aimed to directly assess the trough anticoagulant levels (anti-Xa activities at 90 min after administration) of enoxaparin 0.75 mg/kg versus 0.5 mg/kg in patients undergoing trans-radial coronary angiography (CAG) alone.

**Methods:**

Before CAG, eligible patients were randomly assigned to receive enoxaparin 0.75 mg/kg (High-dose group) or 0.5 mg/kg (Standard-dose group). After CAG, patients undergoing both CAG and PCI were excluded from each group according to the study protocol. Anti-Xa activities were assessed at 0 min, 10 min, and 90 min after enoxaparin was administered. The primary endpoint was anti-Xa activity at 90 min. Target anticoagulation was defined as anti-Xa activities of 0.5-1.8 IU/ml.

**Results:**

A total of 177 patients underwent randomization, 96 of which underwent CAG alone (48 in each group). The baseline characteristics were well balanced between the two groups. In the High-dose compared to the Standard-dose group, (1) the anti-Xa activities were higher at both 90 min (0.80 [0.68, 0.90] IU/ml vs. 0.57 [0.49, 0.69] IU/ml, *p* < 0.001) and 10 min (1.37 [1.16, 1.50] IU/ml vs. 0.94 [0.83, 1.13] IU/ml, *p* < 0.001); (2) the rates of target anticoagulation were higher at 90 min (100.0% [38/38] vs. 72.7% [32/44], *p* < 0.001), although similar at 10 min (100.0% [41/41] vs. 97.9% [46/47], *p* = 1.000).

**Conclusions:**

Enoxaparin 0.75 mg/kg achieved higher anticoagulant levels and higher rates of target anticoagulation at 90 min after administration in patients undergoing trans-radial CAG alone compared to enoxaparin 0.5 mg/kg.

**Trial registration:**

This trial was registered on ClinicalTrials.gov (NCT03145675) on May 21, 2017.

**Supplementary Information:**

The online version contains supplementary material available at 10.1186/s12872-026-05618-x.

## Introduction

Enoxaparin 0.5 mg/kg is one of the guideline-recommended anticoagulant regimens for PCI [1, 2]. It was presumed that this regimen could achieve target anticoagulation (anti-Xa activities of 0.5–1.8 IU/ml) for 90 min. However, it was based on the results of pharmacokinetic simulation rather than direct measurement of the trough anticoagulant levels (anti-Xa activities at 90 min after administration) of enoxaparin [3]. Moreover, no previous study specifically and directly assessed the trough anticoagulant levels of enoxaparin in patients undergoing PCI [4–7]. Furthermore, a pilot study performed in patients undergoing trans-radial coronary angiography (CAG) alone in our center demonstrated that the trough anticoagulant levels were below 0.5 IU/ml (the lower limit of target anticoagulation) in up to 25% of the patients receiving enoxaparin 0.5 mg/kg. Last, The Safety and Efficacy of Enoxaparin in PCI Patients, an International Randomized Evaluation (STEEPLE) study showed that enoxaparin 0.75 mg/kg achieved higher rates of target anticoagulation at both the beginning and the end of PCI (91.8% vs. 78.8%) compared to enoxaparin 0.5 mg/kg [8]. In summary, the trough anticoagulant levels of both enoxaparin 0.75 mg/kg and enoxaparin 0.5 mg/kg may require specific and direct evaluation in patients undergoing PCI.

However, the results of our pilot study raised the concern about the safety of patients undergoing PCI with enoxaparin 0.5 mg/kg, especially if the procedure duration approximated 90 min. Therefore, the Optimizing the Anticoagulant Regimen of Enoxaparin during Percutaneous Coronary Intervention (OPTIENOX-PCI) High-dose study was designed to compare the trough anticoagulant levels of enoxaparin 0.75 mg/kg to those of enoxaparin 0.5 mg/kg specifically in patients undergoing trans-radial CAG alone instead of those undergoing both trans-radial CAG and subsequent PCI, in order to avoid the potentially excessive ischemic risk in patients undergoing both trans-radial CAG and subsequent PCI with enoxaparin 0.5 mg/kg.

## Materials and methods

### Study design and conduct

The OPTIENOX-PCI High-dose was a single-center, prospective, randomized, open-label, active-controlled, parallel, pharmacokinetic study with blinded endpoint assessment. It was conducted in Peking Union Medical College Hospital (PUMCH), Beijing, China. The study protocol was reviewed and approved by the ethics committee in PUMCH (JS-1286). Written informed consent was obtained from each study participant before initiation of any study procedures. The study was conducted in accordance with the principles of the Declaration of Helsinki. It was registered on ClinicalTrials.gov (NCT03145675). The authors were solely responsible for the design and conduct of the study; collection, analysis, and interpretation of the data; and writing, editing, and submitting of the manuscript.

### Study population

The details of the inclusion and the exclusion criteria are shown in Table S1. In brief, patients were eligible for study participation if they were 18 years of age or older, with the diagnoses of stable coronary artery disease or non-ST-segment elevation acute coronary syndromes, who had negative results of cardiac troponin tests within 7 days and planned to undergo elective trans-radial CAG with or without subsequent PCI through successfully inserted radial sheath. Patients with a history of acute myocardial infarction, including ST-segment elevation myocardial infarction or non-ST-segment elevation myocardial infarction, within 30 days, or who had received any fibrinolytic, or anticoagulant, or parenteral antiplatelet agents within 7 days, or who planned to receive any fibrinolytic or antithrombotic agents with the exception of enoxaparin, aspirin, clopidogrel, and ticagrelor during the study period, were excluded from study participation.

### Study procedures and treatment

**Fig. 1:**
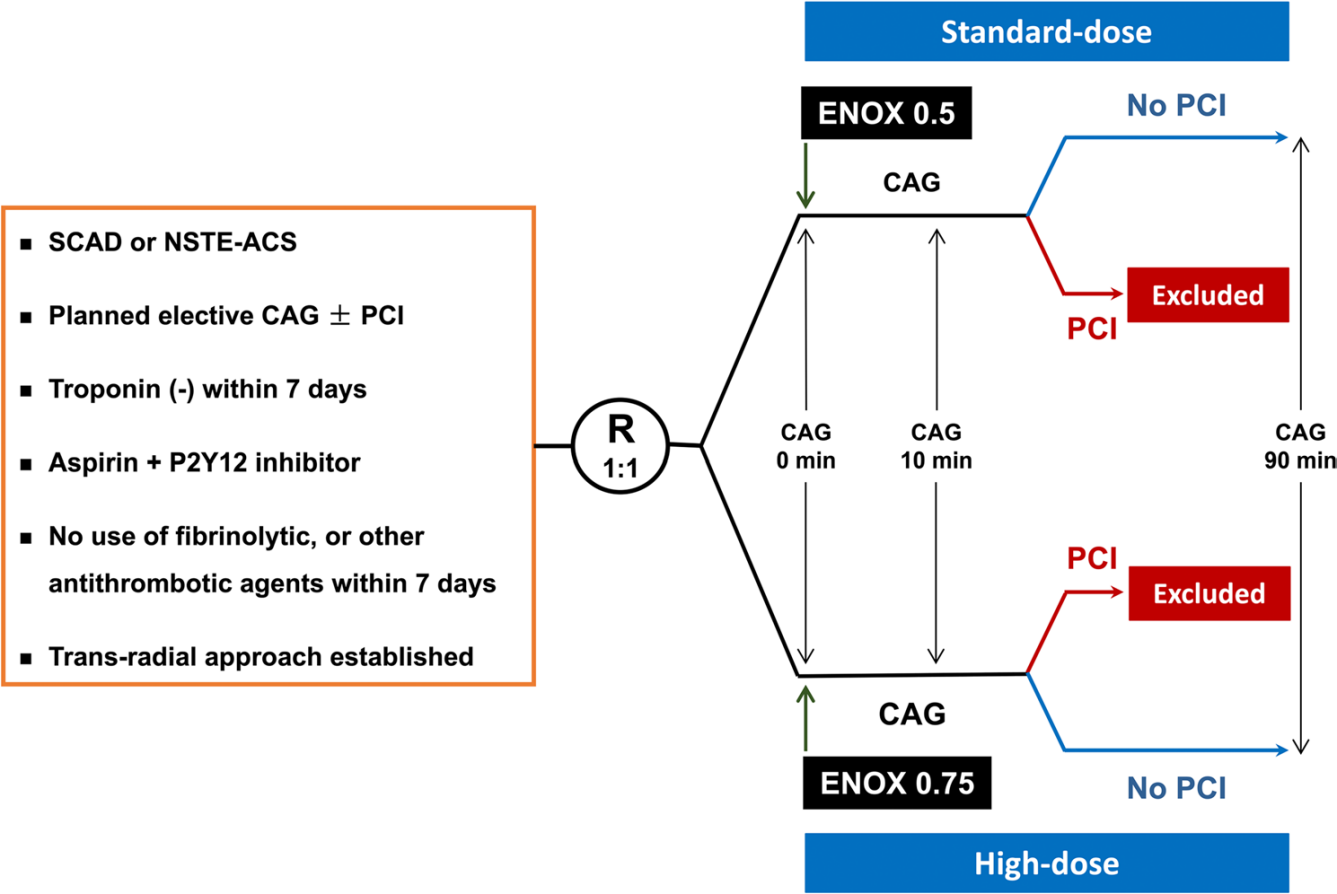
The study design. CAG, coronary angiography; ENOX, enoxaparin; NSTE-ACS, non-ST-segment elevation acute coronary syndrome; PCI, percutaneous coronary intervention; R, randomization; SCAD, stable coronary artery disease

The details of the study procedures and treatment are included in Fig. 1. Briefly, once the radial sheaths were successfully inserted, eligible patients were consecutively, sequentially, and randomly assigned to receive either enoxaparin 0.75 mg/kg (High-dose group) or 0.5 mg/kg (Standard-dose group). The randomization was in a 1:1 ratio, with a block size of 4, and not stratified by any clinical variables. The randomization codes, randomization list, and randomization envelopes were generated, prepared, and sealed, respectively, by a third party who was not involved in the design and conduct of the study. The reproducible randomization codes based on the method of blocked randomization and equal allocation were generated by SAS version 9.4 (SAS Institute, Inc., Cary, NC, USA). The randomization envelopes containing the randomization codes were kept by an independent research assistant, who was solely responsible for the randomization process and otherwise not involved in any study procedure. Once a patient’s eligibility for study participation had been confirmed, the next unopened randomization envelope was opened and the patient was assigned to his/her corresponding treatment group according to the randomization code contained in that randomization envelope. By this means, all eligibility patients were assigned consecutively and in strict sequence as they entered the study, i.e., the next patient to be randomized into the study received the treatment corresponding to the next free number in the randomization list. Once the randomization result of a patient was available, the corresponding dose of enoxaparin was prepared and administered intravenously by a nurse. The assigned regimen of enoxaparin was known only by the research assistant and the nurse, which was not informed to any other cath lab staffs. When the CAG results were available, whether the patients would proceed to subsequent PCI was at the discretion of the procedure operators, who were blinded to the assignment of treatment groups, i.e., the dose of enoxaparin actually given. Patients undergoing CAG alone remained in each group; while those undergoing both CAG and subsequent PCI were excluded from each group due to safety concerns according to the study protocol. The radial sheaths were removed immediately at the end of CAG. No anticoagulant agent was given after CAG. Anti-Xa activities were assessed at 0 min (baseline levels), 10 min (peak levels), and 90 min (trough levels) after enoxaparin was given. The measurements of anti-Xa activities were performed in the central laboratory, the results of which were not available during the procedures. Target anticoagulation was defined as anti-Xa activities of 0.5–1.8 IU/ml, the required anticoagulant levels of enoxaparin for PCI [8]. Over-anticoagulation and under-anticoagulation were defined as anti-Xa activities > 1.8 IU/ml and < 0.5 IU/ml, respectively. The information on cardiac ischemic events and bleeding events was collected at 24 h after randomization.

Although the present study aimed to assess the anticoagulant effect of enoxaparin 0.75 mg/kg compared to that of 0.5 mg/kg in patients undergoing CAG alone, the randomization process was not implemented specifically in patients undergoing CAG alone, which was based on the following considerations: (1) upstream anticoagulation was required to be initiated before the start of trans-radial CAG to prevent thrombotic complications of the radial arteries; (2) patients undergoing CAG alone could only be identified after the results of trans-radial CAG were available. In consequence, if the randomization process was implemented in patients undergoing CAG alone, the study results (anti-Xa activities) would be interfered by the upstream anticoagulation initiated before trans-radial CAG. Therefore, in the present study, the randomization process was performed in all eligible patients before the upstream anticoagulation was initiated according to the study protocol.

### Preparation of enoxaparin

Enoxaparin 60 mg was diluted in normal saline to achieve a final volume of 12 ml, resulting in an enoxaparin solution with a final concentration of 5 mg/ml. In consequence, the regimens of enoxaparin 0.75 mg/kg and 0.5 mg/kg corresponded to the volume of the enoxaparin solution 0.15 ml/kg and 0.1 ml/kg, respectively.

### Blood sampling for measurement of anti-Xa activities

The blood samples for the measurement of anti-Xa activities at 0 min, 10 min, and 90 min (after the procedures) were collected through radial sheaths, catheters, and antecubital veins, respectively, in both groups. The sheaths/catheters were flushed with heparinized saline (unfractionated heparin 50 mg / normal saline 1000 ml) before use. (1) Once the sheaths were inserted, blood was collected through the sheaths before the assigned doses of enoxaparin were intravenously administered. After the first 3 ml of blood was discarded, additional blood was collected with a new syringe for the measurement of anti-Xa activities at 0 min. Then, nitroglycerin 200 ug was injected through the sheaths followed by flushing with 3 ml of heparinized saline. (2) 10 min after enoxaparin was administered, blood was collected through the catheters. After the first 3 ml of blood was discarded, additional blood was collected with a new syringe for the measurement of anti-Xa activities at 10 min. Then, the catheters were flushed with contrast. (3) 90 min after enoxaparin was administered, blood was collected through the antecubital veins for the measurement of anti-Xa activities at 90 min. The blood samples were gently injected or collected into a vacutainer tube containing 3.2% buffered sodium citrate (BD Vacutainer^®^ Citrate Tubes; BD Biosciences, NJ, USA). Then, the tube was filled to capacity and gently inverted 3 to 5 times to ensure complete mixing of the blood with the anticoagulant.

### Processing of blood samples

Processing of blood samples were performed in the central laboratory. All blood samples were centrifuged at 2000 g at 4℃ for 10 min within 1 h of collection, resulting in the plasma for measurement of anti-Xa activities. If feasible, anti-Xa activities were measured within 4 h of sampling; otherwise, the plasma was promptly separated and stored at -70 °C for batch measurement of anti-Xa activities within 2 weeks.

### Measurement of anti-Xa activities

The measurement of anti-Xa activities was performed using a validated chromogenic assay (STA^®^- Liquid Anti-Xa; Diagnostica Stago, Asnières, France), with a lower limit of assay detection of 0.01 IU/ml [3, 5]. The quality of anti-Xa activity determination was assessed by the use of control human plasma containing a predetermined level of low-molecular weight heparin (STA^®^- Quality LMWH; Diagnostica Stago, Asnières, France). The interbatch assay precision (coefficient of variation, %) of the control human plasma was between 3% and 5% during the study period.

### Study endpoints

#### Pharmacokinetic endpoints

The primary endpoint was the anti-Xa activity at 90 min. The secondary endpoints included: (1) the anti-Xa activity at 10 min; (2) the rate of target anticoagulation at 90 min; (3) the rate of target anticoagulation at 10 min.

#### Clinical endpoints

The ischemic endpoints were: (1) major adverse cardiac events (MACEs), i.e., the composite of death, myocardial infarction, urgent coronary revascularization, or definite or probable stent thrombosis; (2) each component of MACEs. The ischemic events were assessed up to 24 h after randomization.

The bleeding endpoints included: (1) major bleeding; (2) major, or minor bleeding; (3) major, minor, or minimal bleeding. Bleeding events were evaluated according to the Thrombolysis in Myocardial Infarction (TIMI) criteria [9] up to 24 h after randomization.

### Statistical analyses

#### Pharmacokinetic analyses

Statistical analyses were performed using SPSS 29.0.1.0 (IBM, Chicago, USA). All randomized patients who received the study medication and underwent CAG alone were defined as the modified intention-to-treat (mITT) population. The primary analyses were performed in the primary analysis population, which was defined as the mITT population but excluding patients who had baseline (0 min) anti-Xa activities ≥ 0.5 IU/ml, i.e., the lower limit of target anticoagulation, before the administration of any study medication. Two-sample t-test or Mann–Whitney U test was applied to compare the anti-Xa activities between the two groups, as appropriate. The rates of target anticoagulation, over-anticoagulation, and under-anticoagulation in each group were compared with Chi-square test or Fisher’s exact test.

To detect the potential bias introduced by excluding patients who had baseline anti-Xa activities ≥ 0.5 IU/ml from the primary analyses, sensitivity analyses were conducted in the entire mITT population.

To confirm the results of the primary analyses, per-protocol analyses were performed in the per-protocol population, i.e., patients in the mITT population who had baseline anti-Xa activities < 0.5 IU/ml and had no major protocol deviation/violation.

In case of the presence of missing anti-Xa activity data, complete case analyses were performed with missing anti-Xa activity data excluded from the dataset. To further evaluate the robustness of the study results, repeat analyses were conducted in the primary analysis population after imputation of missing data through multiple imputation approach (predictive mean matching method) using the mice package in R (version 4.5.2), with 50 imputed datasets generated and the modes selected to replace the missing values.

#### Clinical analyses

The analyses for the ischemic and bleeding endpoints were conducted in the mITT population, which was equivalent to the safety population, i.e., all randomized patients who received the study medication and underwent CAG alone, with Chi-square test or Fisher’s exact test, as appropriate. The analyses for the ischemic endpoints were performed according to the treatment allocation; while the analyses for the bleeding endpoints were conducted based on the treatment actually received.

#### Sample size determination

The present study and the aforementioned pilot study had similar study design, including the inclusion/exclusion criteria. The first 20 patients enrolled in the mITT population of the present study, 12 in the High-dose group and 8 in the Standard-dose group, overlapped with the entire population of the pilot study. Sample size determination of the present study was based on the results of the pilot study since no previous study had reported the anti-Xa activities directly measured at 90 min of enoxaparin administration. However, the enrollment of the rest of the patients in the present study following the pilot study was solely based on the original inclusion/exclusion criteria used in both the pilot study and the present study, which was not interfered by the results of the pilot study.

Based on the results of the pilot study, the anti-Xa activities at 90 min were 0.77 ± 0.17 IU/ml in patients receiving enoxaparin 0.75 mg/kg and 0.57 ± 0.15 IU/ml in those receiving enoxaparin 0.5 mg/kg, respectively. Therefore, 96 (48 per group) patients, with a 2-sided type I error of 0.05, a standard deviation of 0.20 for both groups, and a drop-out rate of 20%, would provide 99% power to detect a difference of 0.20 IU/ml (0.77–0.57 = 0.20) for the anti-Xa activities at 90 min between the two groups. Assuming that 55% of the randomized patients in each group would undergo CAG alone, the required number of patients undergoing randomization would be approximately 175 (96 / 55% = 175).

## Results

### Study population

**Fig. 2:**
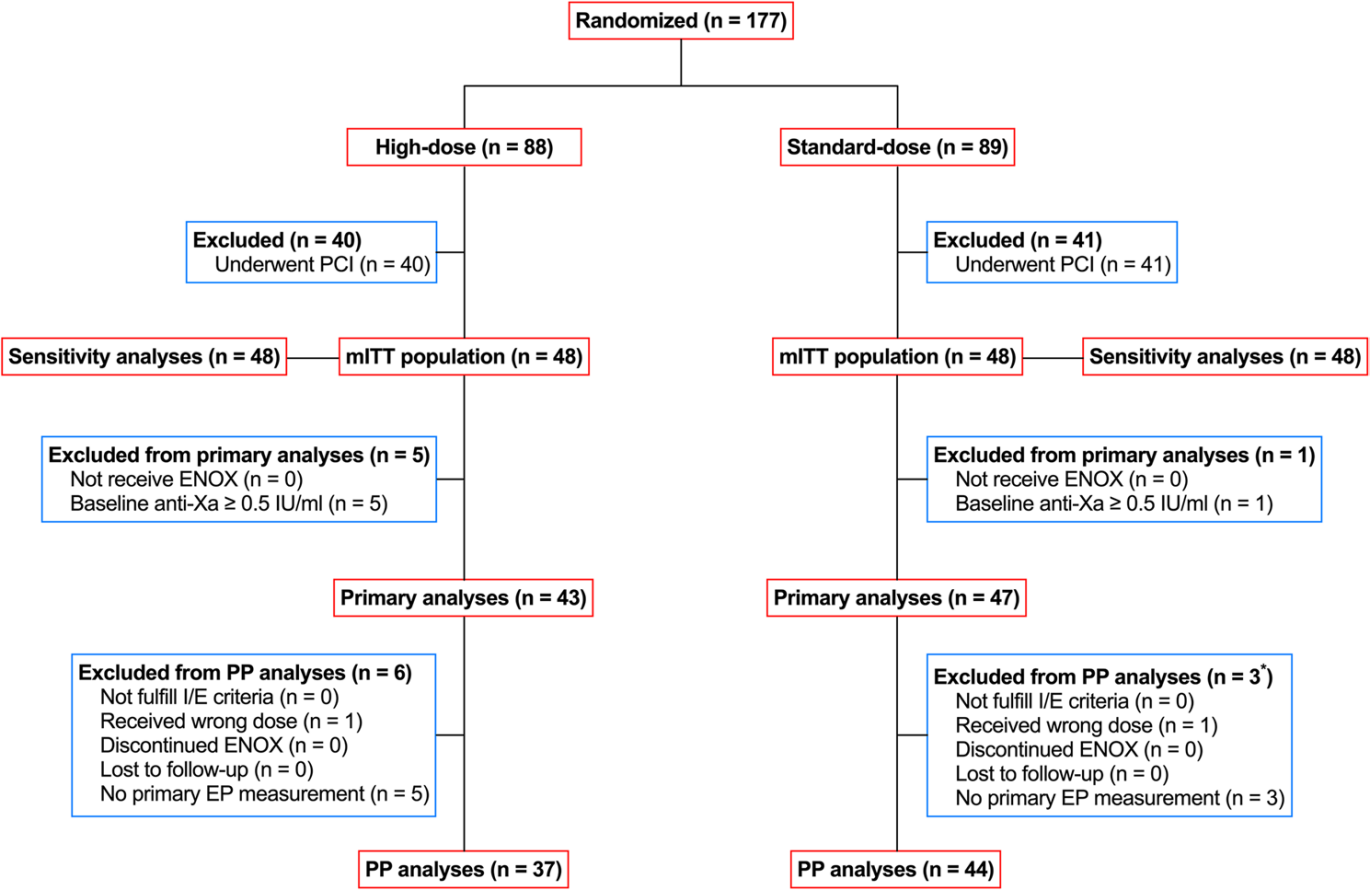
The study flow diagram. ENOX, enoxaparin; EP, endpoint; I/E, inclusion/exclusion; IU, international unit; mITT, modified intention-to-treat; PCI, percutaneous coronary intervention; PP, per-protocol. *Among the 3 patients in the Standard-dose group who were excluded from the PP analyses, 1 patient both received wrong dose of enoxaparin and had no primary endpoint measurement

Between May, 2017 and December, 2018, 177 patients were randomly assigned to the High-dose group (*n* = 88) and the Standard-dose group (*N* = 89), respectively. All patients received the study medication. After excluding patients undergoing both CAG and subsequent PCI (40 from the High-dose group and 41 from the Standard-dose group), those undergoing CAG alone remained in the mITT population (48 in the High-dose group and 48 in the Standard-dose group). Six patients, 5 in the High-dose group and 1 in the Standard-dose group, who had baseline anti-Xa activities ≥ 0.5 IU/ml without identifiable reasons, were excluded from the primary analysis population. Six and 3 patients in the High-dose group and the Standard-dose group, respectively, were further excluded from the per-protocol population due to protocol deviation/violation (Fig. 2). The baseline characteristics were well balanced between the High-dose group and the Standard-dose group in: (1) patients undergoing CAG alone (Table 1); (2) patients undergoing both CAG and subsequent PCI (Table S2); (3) all patients undergoing randomization (Table S3).

**Table 1:** Baseline characteristics (modified intention-to-treat population)

	High-dose (*N* = 48)	Standard-dose (*N* = 48)	*P* value
Demographic characteristics			
Age (years)	63.5 (57.5, 67.0)	65.0 (62.0, 71.0)	0.101
Age ≥ 75 years	3 (6.3%)	4 (8.3%)	1.000
Female	14 (29.2%)	15 (31.3%)	0.824
Body weight (Kg)	70.7 ± 12.7	71.8 ± 11.7	0.641
Body weight < 60 Kg	11 (22.9%)	5 (10.4%)	0.100
Cardiovascular risk factors			
Hypertension	24 (50.0%)	25 (52.1%)	0.838
Diabetes	13 (27.1%)	16 (33.3%)	0.505
Dyslipidemia	15 (31.3%)	15 (31.3%)	1.000
Current smoker	26 (54.2%)	27 (56.3%)	0.837
Family history of premature CAD	14 (29.2%)	14 (29.2%)	1.000
Cardiovascular diseases			
Prior MI	3 (6.3%)	8 (16.7%)	0.109
Prior PCI	9 (18.8%)	12 (25.0%)	0.459
Prior CABG	0 (0.0%)	0 (0.0%)	NA
Prior stroke	9 (18.8%)	6 (12.5%)	0.399
PAD	8 (16.7%)	7 (14.6%)	0.779
CKD	11 (22.9%)	14 (29.2%)	0.485
eGFR (ml/min/1.73m²)^*^	88.9 ± 13.8	82.7 ± 17.7	0.056
Final diagnoses			
Symptomatic CAD	10 (20.8%)	6 (12.5%)	0.273
Stable angina	6 (12.5%)	6 (12.5%)	1.000
Unstable angina	4 (8.3%)	0 (0.0%)	0.125
Asymptomatic CAD	26 (54.2%)	30 (62.5%)	0.408
Coronary atherosclerosis	12 (25.0%)	11 (22.9%)	0.811
Myocardial bridge	1 (2.1%)	3 (6.3%)	0.610
Medications			
Aspirin	48 (100.0%)	48 (100.0%)	NA
P2Y_12_ inhibitor	48 (100.0%)	47 (97.9%)	1.000
Clopidogrel	48 (100.0%)	43 (89.6%)	0.066
Ticagrelor	1 (2.1%)	4 (8.3%)	0.358
β-blocker	29 (60.4%)	32 (66.7%)	0.525
ACEI / ARB / ARNI	22 (45.8%)	25 (52.1%)	0.540
Statin / Ezetimibe / PCSK9 inhibitor	45 (93.8%)	44 (91.7%)	1.000
Oral antidiabetics / Insulin / GLP1-RA	14 (29.2%)	15 (31.3%)	0.824
Procedure characteristics			
Trans-radial approach	48 (100.0%)	48 (100.0%)	NA
Trans-radial alone	47 (97.9%)	48 (100.0%)	1.000
Trans-radial and trans-femoral	1 (2.1%)	0 (0.0%)	1.000
Number of diseased vessels			
1-vessel	7 (14.6%)	8 (16.7%)	0.779
2-vessel	12 (25.0%)	9 (18.8%)	0.459
3-vessel	17 (35.4%)	19 (39.6%)	0.673
Actual time intervals (min)			
Between 0 min and 10 min	10.0 (10.0, 10.0)	10.0 (10.0, 10.0)	0.731
Between 0 min and 90 min	90.0 (90.0, 90.0)	90.0 (90.0, 90.0)	0.692

Values were mean ± standard deviation or median (interquartile range) for continuous data and *n* (%) for categorical data, as appropriate. *P* values were determined using two-sample t-test or Mann–Whitney U test for continuous variables and chi-square test or Fisher’s exact test for categorical variables, as appropriate

*ACEI* angiotensin converting enzyme inhibitor, *ARB *angiotensin receptor blocker, *ARNI *angiotensin receptor-neprilysin inhibitor, *CABG *coronary artery bypass graft, *CAD *coronary artery disease, *CKD *chronic kidney disease, e*GFR *estimated glomerular filtration rate, *GLP1-RA *glucagon-like peptide 1 receptor agonist, *Kg *kilogram, *MI *myocardial infarction, *NA* not applicable, *PAD* peripheral artery disease, *PCI *percutaneous coronary intervention, *PCSK9 *proprotein convertase subtilisin/kexin type 9

^*^eGFR was calculated using the CKD-EPI equation

### Pharmacokinetic results

**Fig. 3:**
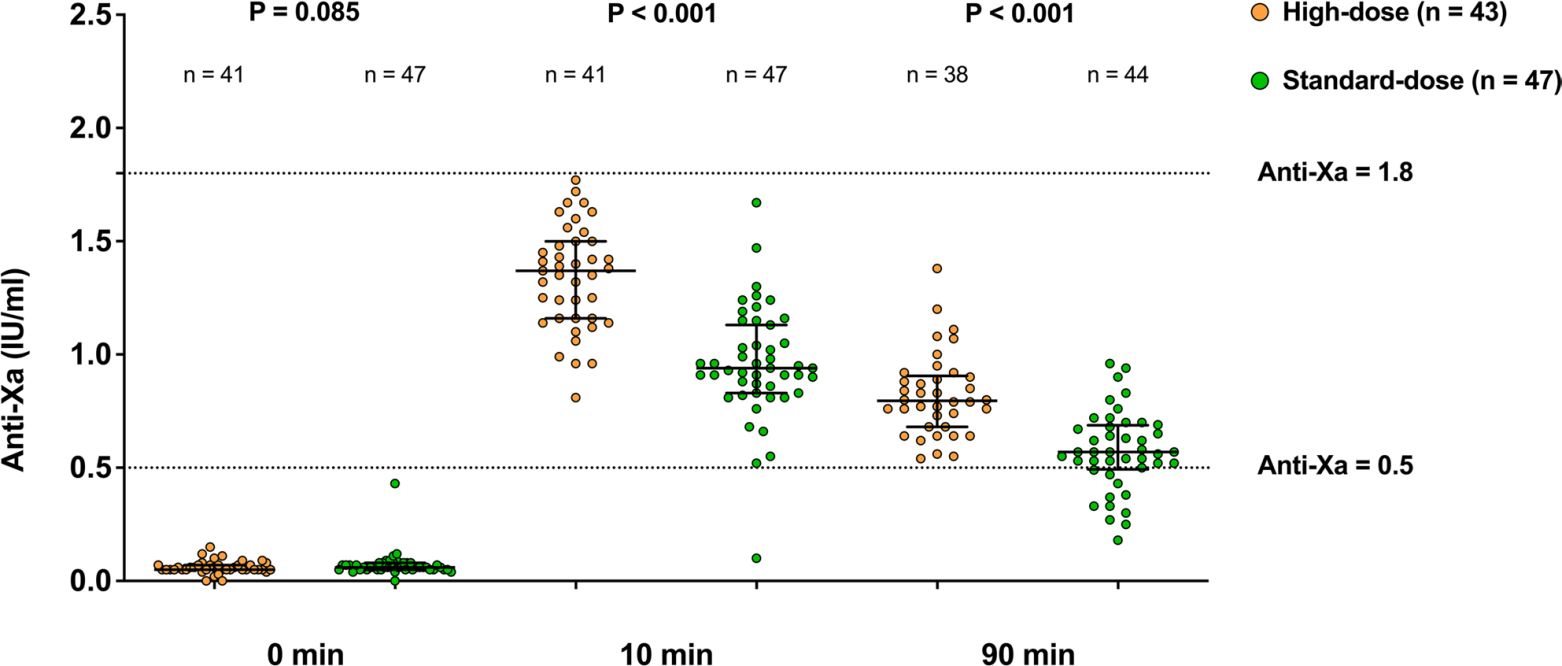
Anti-Xa activities at different time points (primary analysis population). A scatter plot demonstrating the anti-Xa activities at 0 min (immediately before), 10 min, and 90 min after enoxaparin was given in the High-dose group and the Standard-dose group, respectively, in the primary analysis population. *P* values were determined using Mann–Whitney U test. The error bars represented median and interquartile range. The dashed lines indicated anti-Xa activities of 0.5 IU/ml and 1.8 IU/ml, i.e., the lower and upper limits of target anticoagulation, respectively. IU, international unit

The results of the primary analyses are summarized in Table 2; Fig. 3. The primary endpoint, i.e., the anti-Xa activities at 90 min, was 0.80 (0.68, 0.90) IU/ml in the High-dose group compared to 0.57 (0.49, 0.69) IU/ml in the Standard-dose group (*p* < 0.001). The anti-Xa activities at 10 min were 1.37 (1.16, 1.50) IU/ml and 0.94 (0.83, 1.13) IU/ml (*p* < 0.001) in the High-dose group and the Standard-dose group, respectively. The rates of target anticoagulation were higher at 90 min (100.0% [38/38] vs. 72.7% [32/44], *p* < 0.001) in the High-dose group compared to the Standard-dose group, although similar at 10 min (100.0% [41/41] vs. 97.9% [46/47], *p* = 1.000) between the two groups.

**Table 2:** Anti-Xa activities and rates of target, under-, and over-anticoagulation at different time points (primary analysis population)

Time-points	Treatment		*P* value
	High-dose (*N* = 43)	Standard-dose (*N* = 47)	
0 min	0.05 (0.05, 0.07)	0.06 (0.05, 0.08)	0.085
< 0.5 IU/ml	41 (100.0%)	47 (100.0%)	NA
0.5–1.8 IU/ml	0 (0.0%)	0 (0.0%)	NA
> 1.8 IU/ml	0 (0.0%)	0 (0.0%)	NA
10 min	1.37 (1.16, 1.50)	0.94 (0.83, 1.13)	< 0.001
< 0.5 IU/ml	0 (0.0%)	1 (2.1%)	1.000
0.5–1.8 IU/ml	41 (100.0%)	46 (97.9%)	1.000
> 1.8 IU/ml	0 (0.0%)	0 (0.0%)	NA
90 min	0.80 (0.68, 0.90)	0.57 (0.49, 0.69)	< 0.001
< 0.5 IU/ml	0 (0.0%)	12 (27.3%)	< 0.001
0.5–1.8 IU/ml	38 (100.0%)	32 (72.7%)	< 0.001
> 1.8 IU/ml	0 (0.0%)	0 (0.0%)	NA

Target, under-, and over-anticoagulation were defined as anti-Xa activities of 0.5-1.8 IU/ml, < 0.5 IU/ml, and > 1.8 IU/ml, respectively. Values were median (interquartile range) for continuous data and *n* (%) for categorical data, as appropriate. *P* values were determined using Mann–Whitney U test for continuous variables and chi-square test or Fisher’s exact test for categorical variables, as appropriate

*IU* international unit, *NA *not applicable

In the primary analysis population, 5 and 3 patients had missing data of anti-Xa activities in the High-dose group and the Standard-dose group, respectively. The extent and reasons of missingness at different time points were shown in Table S4. The demographic characteristics and final diagnoses were not significantly different between patients with and without missing data of anti-Xa activities in both the High-dose group (Table S5) and the Standard-dose group (Table S6). The results of anti-Xa activities and the rates of target anticoagulation remained stable following missing data imputation (Table S7).

For the sensitivity analyses conducted in the mITT population (Table S8, Fig. S1), the anti-Xa activities at 90 min was 0.79 (0.64, 0.92) IU/ml in the High-dose group compared to 0.57 (0.49, 0.69) IU/ml in the Standard-dose group (*p* < 0.001). The rates of target anticoagulation at 90 min were 100.0% (43/43) and 73.3% (33/45) in the High-dose group and the Standard-dose group, respectively (*p* < 0.001). The results of the per-protocol analyses (Table S9, Fig. S2) were also consistent with those of the primary analyses.

### Clinical outcomes

No MACEs, each component of MACEs, TIMI major bleeding, or TIMI minor bleeding occurred in both groups during the first 24 h after randomization. Two (4.2%) and 4 (8.3%) patients had TIMI minimal bleeding in the High-dose group and the Standard-dose group, respectively (*p* = 0.673) (Table 3).

**Table 3:** Clinical events within 24 hours (modified intention-to-treat population)

Clinical events	Treatment		*P* value
	High-dose (*N* = 48)	Standard-dose (*N* = 48)	
MACEs	0 (0.0%)	0 (0.0%)	NA
Major bleeding	0 (0.0%)	0 (0.0%)	NA
Minor bleeding	0 (0.0%)	0 (0.0%)	NA
Minimal bleeding	2 (4.2%)	4 (8.3%)	0.673
Major or minor bleeding	0 (0.0%)	0 (0.0%)	NA
Major or minor or minimal bleeding	2 (4.2%)	4 (8.3%)	0.673

Values were presented as *n* (%). P values were determined using chi-square test or Fisher’s exact test, as appropriate

*MACE* major adverse cardiac events, *NA *not applicable, *TIMI *Thrombolysis in Myocardial Infarction

## Discussion

The present study demonstrated that in patients undergoing trans-radial CAG alone, (1) enoxaparin 0.75 mg/kg achieved higher anti-Xa activities and higher rates of target anticoagulation at 90 min after administration compared to enoxaparin 0.5 mg/kg; (2) the rates of target anticoagulation were similar between the two regimens at 10 min after administration; (3) the rates for ischemic and bleeding events were low in both groups within 24 h of randomization.

The present study specifically and directly investigated the anticoagulant levels of enoxaparin 0.75 mg/kg versus 0.5 mg/kg at both 90 min (trough levels) and 10 min (peak levels) after administration in patients undergoing CAG alone. Based on the study results, the anti-Xa activities at 90 min were below the lower limit of target anticoagulation in as much as 27.3% (12/44) of the patients with enoxaparin 0.5 mg/kg, although the median value of the anti-Xa activities at 90 min (0.57 [0.49, 0.69]) was still above 0.5 IU/ml. While, target anticoagulation was maintained in 100.0% (38/38) of patients receiving enoxaparin 0.75 mg/kg compared to only 72.7% (32/44) of those receiving enoxaparin 0.5 mg/kg at 90 min (*p* < 0.001), although both groups achieved high rates of target anticoagulation at 10 min (100.0% [41/41] vs. 97.9% [46/47], *p* = 1.000). In summary, enoxaparin 0.75 mg/kg was a better anticoagulation regimen than enoxaparin 0.5 mg/kg in terms of providing adequate anticoagulation for 90 min at least in patients undergoing CAG alone.

Patients undergoing PCI usually carry higher thrombotic risk compared to those undergoing CAG alone, due to longer procedure duration, higher contrast load, catheter manipulation, endothelial injury, et al. Therefore, achieving and maintaining target anticoagulation during the entire course of PCI is essential to prevent peri-procedural thrombotic and bleeding complications. Under- and over-anticoagulation are associated with increased ischemic and bleeding risk, respectively [10–13]. Although conducted in patients undergoing CAG alone, the present study might provide important implications for those undergoing PCI. First, if enoxaparin 0.5 mg/kg did not provide adequate anticoagulant levels at 90 min in patients undergoing CAG alone, it would not have done so in patients undergoing PCI, too. Second, the study results at least would not have underestimated the rate of target anticoagulation or overestimated the rate of under-anticoagulation at 90 min in PCI patients receiving enoxaparin 0.5 mg/kg. Third, given the association between under-anticoagulation and increased thrombotic risk [10–13], enoxaparin 0.5 mg/kg might not adequately prevent thrombotic events for patients undergoing PCI, especially if the procedure duration approximated 90 min. Fourth, although the present study could not confidently justify the use of enoxaparin 0.75 mg/kg for PCI, at least it may indicate that enoxaparin 0.75 mg/kg would provide more confident anticoagulant levels for 90 min in patients undergoing PCI compared to enoxaparin 0.5 mg/kg. In summary, the results of the present study would not overestimate the limitations of enoxaparin 0.5 mg/kg and the advantage of enoxaparin 0.75 mg/kg when used in patients undergoing PCI. Of course, we do acknowledge that all the above speculations need to be confirmed with dedicate studies comparing enoxaparin 0.75 mg/kg and 0.5 mg/kg specifically and directly in patients undergoing PCI.

Higher anticoagulant levels, such as those achieved with enoxaparin 0.75 mg/kg compared to enoxaparin 0.5 mg/kg, might be associated with increased bleeding risk [10]. The STEEPLE study did demonstrate that the primary safety endpoint, i.e., the rate of the protocol-defined non-CABG-related major or minor bleeding, was numerically higher in patients receiving enoxaparin 0.75 mg/kg compared to those receiving enoxaparin 0.5 mg/kg (event rates: 6.5% vs. 5.9%, absolute difference: + 0.6%). However, the rates of the protocol-defined non-CABG-related major bleeding were similar between the two enoxaparin groups (1.2% vs. 1.2%, absolute difference: 0%) in the STEEPLE study, indicating that the risk of severe bleeding was comparable between patients receiving enoxaparin 0.75 mg/kg and those receiving enoxaparin 0.5 mg/kg. Furthermore, in the STEEPLE study, all patients underwent PCI via trans-femoral approach and approximately 40% of the patients underwent PCI with glycoprotein IIb/IIIa inhibitors. Both trans-femoral approach and glycoprotein IIb/IIIa inhibitors, which are associated with increased bleeding risk in patients undergoing PCI [14–21], have been used less frequently with the advent of trans-radial approach and more potent oral P2Y12 inhibitors [1, 2, 21–25]. In consequence, the absolute difference in the bleeding risk between enoxaparin 0.75 mg/kg and 0.5 mg/kg would be even less along with the reduction of the overall bleeding risk in patients undergoing PCI in contemporary era. In summary, the potential increase in bleeding risk with enoxaparin 0.75 mg/kg compared to 0.5 mg/kg would not be a significant concern, especially under the background of contemporary PCI strategies. Of course, we acknowledge that the rates of bleeding events in the present study was low, which was underpowered to provide definite conclusion regarding the safety of enoxaparin 0.75 mg/kg compared to enoxaparin 0.5 mg/kg.

### Limitations

The results of the present study should be interpreted with caution due to several limitations.

First, only 177 patients were enrolled in the study and only 48 patients undergoing CAG alone in each group were included in the primary analyses. However, this sample size was adequate enough to provide more than 99% power with a 2-sided alpha level of 0.05 to detect the difference of 0.23 IU/ml (0.80–0.57 = 0.23) for the primary endpoint, although it is underpowered to detect the difference in clinical endpoints between the two groups.

Second, although the patients were randomly assigned to receive either of the two regimens of enoxaparin before CAG, whether the patients would undergo subsequent PCI was at the discretion of the procedure operators and based on the results of CAG. Therefore, excluding patients undergoing subsequent PCI after randomization might introduce selection bias. However, the procedure operators, who made the decision on whether to perform subsequent PCI, were blinded to the assignment of the study treatment. In addition, the baseline characteristics were well balanced between the two groups not only in patients undergoing CAG alone, but also in patients undergoing both CAG and subsequent PCI, as well as in all patients undergoing randomization, although unmeasured confounders might still be present, especially when the per-group sample sizes were small.

Third, the open-label design of the study may introduce observer and/or reporting bias. However, the primary endpoint of the present study was the objective results of anti-Xa assay and the pharmacokinetic operator was blinded to the assignment of the study treatment.

Fourth, the blood samples for the measurement of anti-Xa activities were collected with different approaches, including radial sheaths, catheters, and antecubital veins, which might also introduce measurement bias. However, comparing the anti-Xa activities of blood samples collected with different approaches might be acceptable according to the methods used in previous studies [3, 5]. Moreover, the sample collection routes were similar between the two groups at each sampling time point, which might not interfere with the between-group comparison results of anti-Xa activities.

Fifth, excluding patients who had baseline anti-Xa activities ≥ 0.5 IU/ml from the primary analyses may introduce bias and underestimate the variance of anti-Xa activities in the study population. Nevertheless, the results of the sensitivity analyses conducted in the entire mITT population were consistent with those of the primary analyses, which might mitigate this concern.

Sixth, the study was performed in patients undergoing CAG alone aiming to avoid potential under-anticoagulation and thrombotic risk in patients undergoing both CAG and subsequent PCI with enoxaparin 0.5 mg/kg. As mentioned, patients undergoing PCI usually carry higher thrombotic risk and need prolonged anticoagulation compared to those undergoing CAG alone. Therefore, the study results might not be fully generalizable to patients undergoing PCI.

Seventh, the study results might not be extrapolated to patients with acute myocardial infarction, which was an exclusion criterion of the present study.

Last, we must acknowledge that the present study could not assess the clinical efficacy and safety of enoxaparin 0.75 mg/kg versus 0.5 mg/kg considering the small sample size, the short duration of follow-up, and the low event rates of the clinical endpoints.

## Conclusions

Enoxaparin 0.75 mg/kg achieved higher anticoagulant levels and higher rates of target anticoagulation at 90 min after administration in patients undergoing trans-radial CAG alone compared to enoxaparin 0.5 mg/kg. However, randomized and adequately powered outcome studies are required to further evaluate the anticoagulant levels as well as the clinical efficacy and safety of enoxaparin 0.75 mg/kg versus 0.5 mg/kg specifically and directly in patients undergoing PCI. 

## Supplementary Information

Supplementary Material 1.

Supplementary Material 2.

Supplementary Material 3.

## Data Availability

The datasets used and/or analysed during the current study are available from the corresponding author on reasonable request.

## Abbreviations

CABG: Coronary artery bypass graft
CAG: Coronary angiography
IU: International unit
MACE: Major adverse cardiac event
mITT: Modified intention-to-treat
OPTIENOX-PCI: Optimizing the Anticoagulant Regimen of Enoxaparin during Percutaneous Coronary Intervention
PCI: Percutaneous coronary intervention
STEEPLE: The Safety and Efficacy of Enoxaparin in PCI Patients, an International Randomized Evaluation
TIMI: Thrombolysis in Myocardial Infarction
UFH: Unfractionated heparin
