# Supplementary material for: Trough anticoagulant levels of high-dose versus standard-dose intravenous enoxaparin in patients undergoing trans-radial coronary angiography alone
Source: BMC Cardiovasc Disord. 2026 Feb 19;26:257. doi: 10.1186/s12872-026-05618-x (PMC13019850; doi:10.1186/s12872-026-05618-x)
Supplement: Supplementary file 1 — Supplementary Material 1. [file 12872_2026_5618_MOESM1_ESM.docx]

**Supplementary Figures**

**
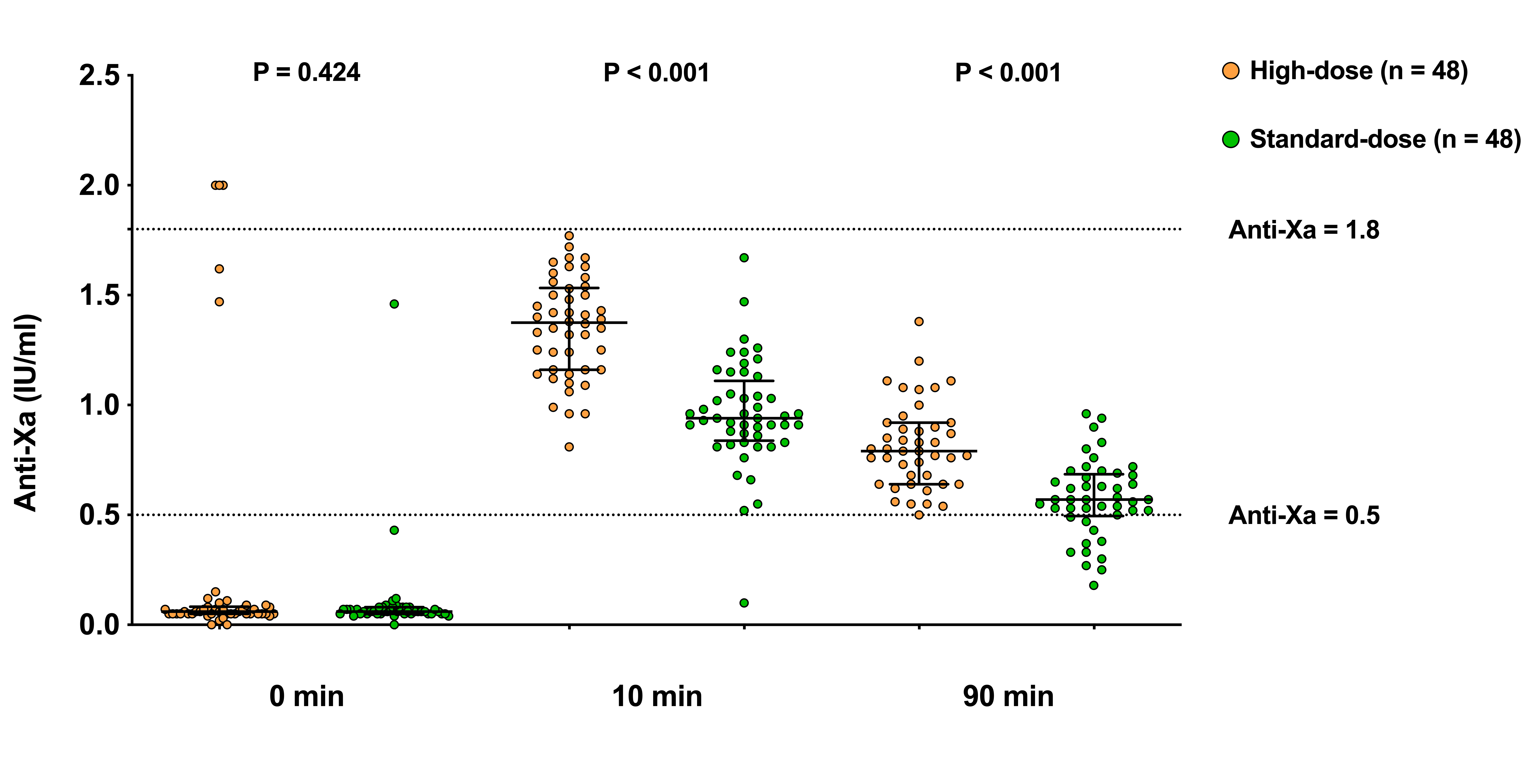
**

**Figure S1. Anti-Xa activities at different time points (modified intention-to-treat population).**

A scatter plot demonstrating the anti-Xa activities at 0 min (immediately before), 10 min, and 90 min after enoxaparin was given in the High-dose group and the Standard-dose group, respectively, in the modified intention-to-treat population. P values were determined using Mann–Whitney U test. The error bars represented median and interquartile range. The dashed lines indicated anti-Xa activities of 0.5 IU/ml and 1.8 IU/ml, i.e., the lower and upper limits of target anticoagulation, respectively.

IU, international unit.


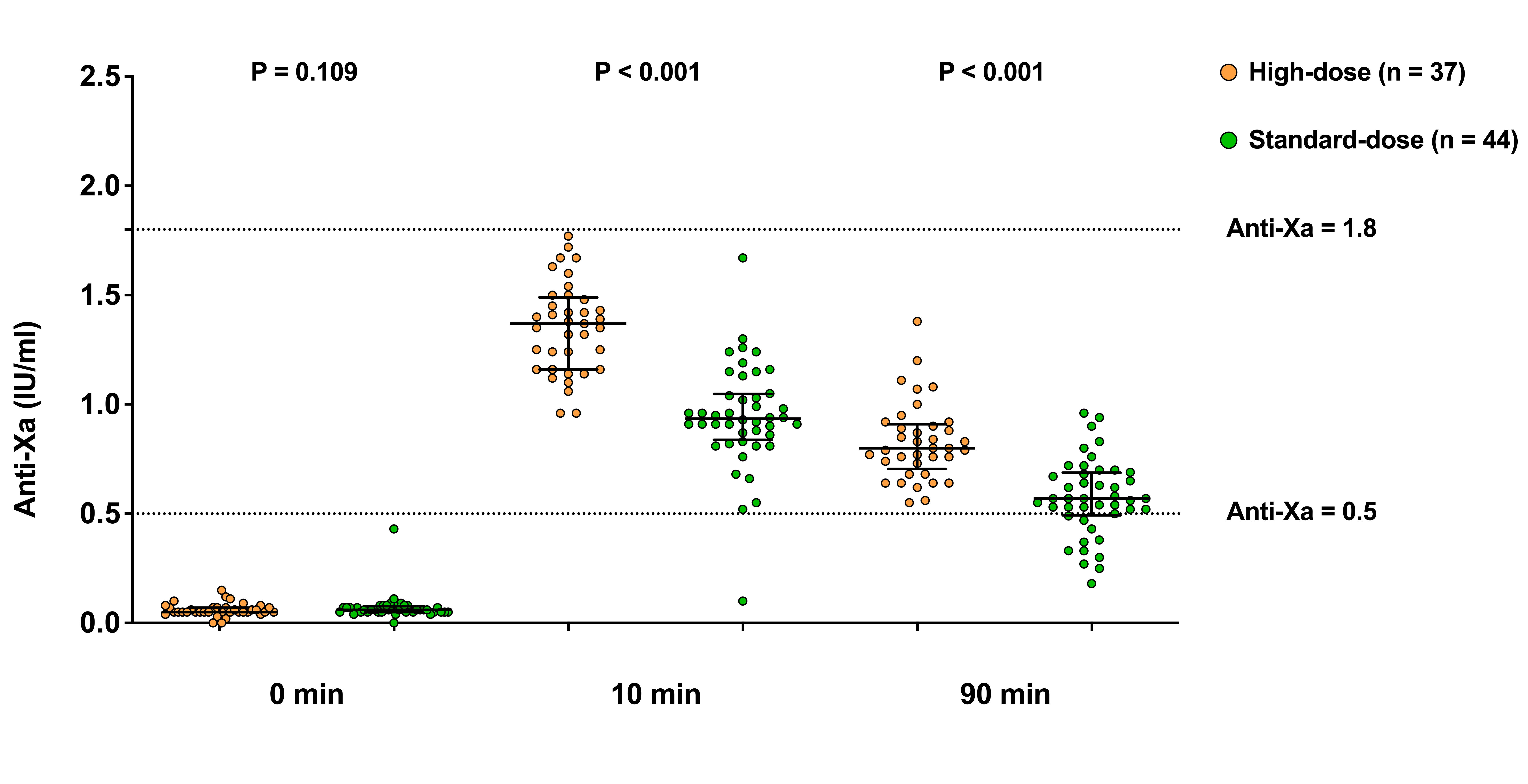


**Figure S2. Anti-Xa activities at different time points (per-protocol population).**

A scatter plot demonstrating the anti-Xa activities at 0 min (immediately before), 10 min, and 90 min after enoxaparin was given in the High-dose group and the Standard-dose group, respectively, in the per-protocol population. P values were determined using Mann–Whitney U test. The error bars represented median and interquartile range. The dashed lines indicated anti-Xa activities of 0.5 IU/ml and 1.8 IU/ml, i.e., the lower and upper limits of target anticoagulation, respectively.

IU, international unit.
