# Supplementary material for: Trough anticoagulant levels of high-dose versus standard-dose intravenous enoxaparin in patients undergoing trans-radial coronary angiography alone
Source: BMC Cardiovasc Disord. 2026 Feb 19;26:257. doi: 10.1186/s12872-026-05618-x (PMC13019850; doi:10.1186/s12872-026-05618-x)
Supplement: Supplementary file 2 — Supplementary Material 2. [file 12872_2026_5618_MOESM2_ESM.docx]

**Supplementary Tables**

**Table S1. Inclusion and exclusion criteria**

| **Inclusion Criteria:** |
| --- |
| 1. Provision of written informed consent. |
| 2. Aged 18 years or older, male or female. |
| 3. Documented stable coronary artery disease or non-ST-segment elevation acute coronary syndromes. |
| 4. Plan to undergo elective trans-radial coronary angiography with or without subsequent percutaneous coronary intervention (PCI). |
| 5. No fibrinolytic, or anticoagulant, or parenteral antiplatelet therapy within 7 days of screening. |
| 6. Negative cardiac troponin test within 7 days of screening. |
| 7. Trans-radial approach successfully established. |
| 8. Females who are either post-menopausal > 1 year or surgically sterile. |
|  |
| **Exclusion Criteria:** |
| 1. Recent (within 30 days of screening) acute myocardial infarction, including ST-segment elevation myocardial infarction or non-ST-segment elevation myocardial infarction. |
| 2. The aim of the index coronary angiography is to undergo primary PCI or early PCI for acute coronary syndromes. |
| 3. Any indications other than coronary artery disease (e.g., atrial fibrillation, prosthetic heart valve, venous thromboembolism, ventricular thrombosis, et al) for fibrinolytic or anticoagulant treatment during study period. |
| 4. Planned use of any fibrinolytic or antithrombotic agents, with the exception of enoxaparin, aspirin, clopidogrel, and ticagrelor during study period. |
| 5. Planned coronary artery bypass graft during study period. |
| 6. Increased bleeding risk, including  • any history of intracranial, intraocular, retroperitoneal, or spinal bleeding;  • recent (within 30 days of screening) gastrointestinal bleeding;  • recent (within 30 days of screening) major trauma or major surgery;  • planned surgery or other invasive procedure during study period;  • sustained uncontrolled hypertension (systolic blood pressure > 180 mmHg or diastolic blood pressure > 100 mmHg) within 7 days of screening;  • history of hemorrhagic disorders, e.g., haemophilia, von Willebrand’s disease;  • inability to discontinue non-steroidal anti-inflammatory drugs during study period;  • platelet count less than 100,000/mm^3^ or hemoglobin < 10 g/dL within 7 days of screening. |
| 7. Contraindications for enoxaparin, e.g., hypersensitivity, active bleeding, bleeding diathesis, coagulation disorders, acute infectious endocarditis, thrombocytopenia (including heparin-induced thrombocytopenia), cerebral hemorrhage, severe liver of kidney diseases, severe hypertension, stroke, retinopathy, et al. |
| 8. History of intolerance to enoxaparin. |
| 9. Patient requires dialysis or has a creatinine clearance (CrCl) < 30 mL/min as calculated by the Cockcroft-Gault equation: CrCl = (140 - Age) × WT / (72 × SCr) (× 0.85 for females), where WT is weight in kg, SCr is serum creatinine in mg/dL measured within 7 days of screening. |
| 10. Any acute or chronic unstable conditions in the past 30 days which, in the opinion of the investigators, may either put the patient at risk or influence the result of the study, e.g., active cancer, et al. |
| 11. Any condition that may increase the risk of non-compliance to study protocol or follow-up, e.g., history of drug addiction or alcohol abuse, et al. |
| 12. Patients who has previously been randomized in this study. |
| 13. Participation in another investigational drug or device study within 30 days of screening. |
| 14. Involvement in the planning and conduct of the study (applies to investigators, contract research organization staffs, and study site staffs). |
| 15. Known pregnancy, breast-feeding, or intend to become pregnant during the study period. |
| 16. Any condition which in the opinion of the investigators would make it unsafe or unsuitable for the patient to participate in this study. |

PCI, percutaneous coronary intervention.

**Table S2. Baseline characteristics (patients undergoing both CAG and subsequent PCI)**

|  | **High-dose**  **(N = 40)** | **Standard-dose**  **(N = 41)** | ***P* value** | |
| --- | --- | --- | --- | --- |
| Demographic characteristics | | | |  |
| Age (years) | 64.00 (58.25, 68.00) | 59.00 (50.50, 68.00) | 0.100 | |
| Age ≥ 75 years | 1 (2.5%) | 3 (7.3%) | 0.626 | |
| Female | 11 (27.5%) | 8 (19.5%) | 0.396 | |
| Body weight (Kg) | 70.7 ± 10.0 | 73.7 ± 11.6 | 0.222 | |
| Body weight < 60 Kg | 3 (7.5%) | 5 (12.2%) | 0.737 | |
| Cardiovascular risk factors | | | |  |
| Hypertension | 22 (55.0%) | 28 (68.3%) | 0.218 | |
| Diabetes | 12 (30.0%) | 16 (39.0%) | 0.393 | |
| Dyslipidemia | 13 (32.5%) | 15 (36.6%) | 0.699 | |
| Current smoker | 21 (52.5%) | 23 (56.1%) | 0.745 | |
| Family history of premature CAD | 12 (30.0%) | 11 (26.8%) | 0.752 | |
| Cardiovascular diseases | | | |  |
| Prior MI | 6 (15.0%) | 4 (9.8%) | 0.704 | |
| Prior PCI | 11 (27.5%) | 9 (22.0%) | 0.563 | |
| Prior CABG | 0 (0.0%) | 1 (2.4%) | 1.000 | |
| Prior stroke | 3 (7.5%) | 7 (17.1%) | 0.331 | |
| PAD | 7 (17.5%) | 4 (9.8%) | 0.309 | |
| CKD | 14 (35.0%) | 10 (24.4%) | 0.296 | |
| eGFR (ml/min/1.73m²)^*^ | 89.6 ± 11.8 | 88.5 ± 15.2 | 0.715 | |
| Final diagnoses | | | |  |
| Symptomatic CAD | 23 (57.5%) | 31 (75.6%) | 0.084 | |
| Stable angina | 8 (20.0%) | 16 (39.0%) | 0.061 | |
| Unstable angina | 15 (37.5%) | 15 (36.6%) | 0.932 | |
| Asymptomatic CAD | 17 (42.5%) | 10 (24.4%) | 0.084 | |
| Myocardial bridge | 0 (0.0%) | 0 (0.0%) | NA | |
| Medications | | | |  |
| Aspirin | 40 (100.0%) | 41 (100.0%) | NA | |
| P2Y_12_ inhibitor | 40 (100.0%) | 41 (100.0%) | NA | |
| Clopidogrel | 38 (95.0%) | 38 (92.7%) | 1.000 | |
| Ticagrelor | 4 (10.0%) | 3 (7.3%) | 0.973 | |
| β-blocker | 30 (75.0%) | 30 (73.2%) | 0.851 | |
| ACEI / ARB / ARNI | 21 (52.5%) | 26 (63.4%) | 0.320 | |
| Statin / Ezetimibe / PCSK9 inhibitor | 38 (95.0%) | 39 (95.1%) | 1.000 | |
| Oral antidiabetics / Insulin / GLP1-RA | 13 (32.5%) | 17 (41.5%) | 0.404 | |
| Procedure characteristics | | | |  |
| Trans-radial approach | 40 (100.0%) | 41 (100.0%) | NA | |
| Trans-radial alone | 36 (90.0%) | 38 (92.7%) | 0.973 | |
| Trans-radial and trans-femoral | 4 (10.0%) | 3 (7.3%) | 0.973 | |
| Number of diseased vessels |  |  |  | |
| 1-vessel | 11 (27.5%) | 8 (19.5%) | 0.396 | |
| 2-vessel | 11 (27.5%) | 18 (43.9%) | 0.124 | |
| 3-vessel | 18 (45.0%) | 15 (36.6%) | 0.441 | |

ACEI, angiotensin converting enzyme inhibitor; ARB, angiotensin receptor blocker; ARNI, angiotensin receptor-neprilysin inhibitor; CABG, coronary artery bypass graft; CAD, coronary artery disease; CAG, coronary angiography; CKD, chronic kidney disease; eGFR, estimated glomerular filtration rate; GLP1-RA, glucagon-like peptide 1 receptor agonist; Kg, kilogram; MI, myocardial infarction; NA, not applicable; PAD, peripheral artery disease; PCI, percutaneous coronary intervention; PCSK9, proprotein convertase subtilisin/kexin type 9.

^*^eGFR was calculated using the CKD-EPI equation.

Note: Values were mean ± standard deviation or median (interquartile range) for continuous data and n (%) for categorical data, as appropriate. P values were determined using two-sample t-test or Mann–Whitney U test for continuous variables and chi-square test or Fisher’s exact test for categorical variables, as appropriate.

**Table S3. Baseline characteristics (all patients undergoing randomization)**

|  | **High-dose**  **(N = 88)** | **Standard-dose**  **(N = 89)** | ***P* value** |
| --- | --- | --- | --- |
| Demographic characteristics | | | |
| Age (years) | 64.00 (58.25, 67.75) | 63.00 (54.00, 70.00) | 0.911 |
| Age ≥ 75 years | 4 (4.5%) | 7 (7.9%) | 0.360 |
| Female | 25 (28.4%) | 23 (25.8%) | 0.701 |
| Body weight (Kg) | 70.7 ± 11.5 | 72.7 ± 11.6 | 0.252 |
| Body weight < 60 Kg | 14 (15.9%) | 10 (11.2%) | 0.364 |
| Cardiovascular risk factors | | | |
| Hypertension | 46 (52.3%) | 53 (59.6%) | 0.329 |
| Diabetes | 25 (28.4%) | 32 (36.0%) | 0.283 |
| Dyslipidemia | 28 (31.8%) | 30 (33.7%) | 0.789 |
| Current smoker | 47 (53.4%) | 50 (56.2%) | 0.711 |
| Family history of premature CAD | 26 (29.5%) | 25 (28.1%) | 0.831 |
| Cardiovascular diseases | | | |
| Prior MI | 9 (10.2%) | 12 (13.5%) | 0.503 |
| Prior PCI | 20 (22.7%) | 21 (23.6%) | 0.891 |
| Prior CABG | 0 (0.0%) | 1 (1.1%) | 1.000 |
| Prior stroke | 12 (13.6%) | 13 (14.6%) | 0.853 |
| PAD | 15 (17.0%) | 11 (12.4%) | 0.379 |
| CKD | 25 (28.4%) | 24 (27.0%) | 0.830 |
| eGFR (ml/min/1.73m²)^*^ | 89.2 ± 12.9 | 85.3 ± 16.9 | 0.086 |
| Final diagnoses | | | |
| Symptomatic CAD | 33 (37.5%) | 37 (41.6%) | 0.580 |
| Stable angina | 14 (15.9%) | 22 (24.7%) | 0.145 |
| Unstable angina | 19 (21.6%) | 15 (16.9%) | 0.424 |
| Asymptomatic CAD | 43 (48.9%) | 40 (44.9%) | 0.601 |
| Coronary atherosclerosis | 12 (13.6%) | 11 (12.4%) | 0.801 |
| Myocardial bridge | 1 (1.1%) | 3 (3.4%) | 0.621 |
| Medications | | | |
| Aspirin | 88 (100.0%) | 89 (100.0%) | NA |
| P2Y_12_ inhibitor | 88 (100.0%) | 88 (98.9%) | 1.000 |
| Clopidogrel | 86 (97.7%) | 81 (91.0%) | 0.108 |
| Ticagrelor | 5 (5.7%) | 7 (7.9%) | 0.563 |
| β-blocker | 59 (67.0%) | 62 (69.7%) | 0.708 |
| ACEI / ARB / ARNI | 43 (48.9%) | 51 (57.3%) | 0.261 |
| Statin / Ezetimibe / PCSK9 inhibitor | 83 (94.3%) | 83 (93.3%) | 0.770 |
| Oral antidiabetics / Insulin / GLP1-RA | 27 (30.7%) | 32 (36.0%) | 0.457 |
| Procedure characteristics | | | |
| Trans-radial approach | 88 (100.0%) | 89 (100.0%) | NA |
| Trans-radial alone | 83 (94.3%) | 86 (96.6%) | 0.705 |
| Trans-radial and trans-femoral | 5 (5.7%) | 3 (3.4%) | 0.705 |
| Number of diseased vessels |  |  |  |
| 1-vessel | 18 (20.5%) | 16 (18.0%) | 0.676 |
| 2-vessel | 23 (26.1%) | 27 (30.3%) | 0.535 |
| 3-vessel | 35 (39.8%) | 34 (38.2%) | 0.830 |

ACEI, angiotensin converting enzyme inhibitor; ARB, angiotensin receptor blocker; ARNI, angiotensin receptor-neprilysin inhibitor; CABG, coronary artery bypass graft; CAD, coronary artery disease; CKD, chronic kidney disease; eGFR, estimated glomerular filtration rate; GLP1-RA, glucagon-like peptide 1 receptor agonist; Kg, kilogram; MI, myocardial infarction; NA, not applicable; PAD, peripheral artery disease; PCI, percutaneous coronary intervention; PCSK9, proprotein convertase subtilisin/kexin type 9.

^*^eGFR was calculated using the CKD-EPI equation.

Note: Values were mean ± standard deviation or median (interquartile range) for continuous data and n (%) for categorical data, as appropriate. P values were determined using two-sample t-test or Mann–Whitney U test for continuous variables and chi-square test or Fisher’s exact test for categorical variables, as appropriate.

**Table S4 Summary of missing data of anti-Xa activities at different time points (primary analysis population)**

| **Time-points** | **Reasons for missing data** | **Treatment** | | ***P* value** |
| --- | --- | --- | --- | --- |
|  |  | **High-dose**  **(N = 43)** | **Standard-dose**  **(N = 47)** |  |
| 0 min | All reasons | 2 (4.7%) | 0 (0.0%) | 0.225 |
|  | Unknown | 2 (4.7%) | 0 (0.0%) | 0.225 |
|  | No sampling | 0 (0.0%) | 0 (0.0%) | NA |
|  | Sample loss | 0 (0.0%) | 0 (0.0%) | NA |
|  | Sample hemolysis | 0 (0.0%) | 0 (0.0%) | NA |
| 10 min | All reasons | 2 (4.7%) | 0 (0.0%) | 0.225 |
|  | Unknown | 2 (4.7%) | 0 (0.0%) | 0.225 |
|  | No sampling | 0 (0.0%) | 0 (0.0%) | NA |
|  | Sample loss | 0 (0.0%) | 0 (0.0%) | NA |
|  | Sample hemolysis | 0 (0.0%) | 0 (0.0%) | NA |
| 90 min | All reasons | 5 (11.6%) | 3 (6.4%) | 0.615 |
|  | Unknown | 2 (4.7%) | 1 (2.1%) | 0.938 |
|  | No sampling | 1 (2.3%) | 0 (0.0%) | 0.478 |
|  | Sample loss | 1 (2.3%) | 0 (0.0%) | 0.478 |
|  | Sample hemolysis | 1 (2.3%) | 2 (4.3%) | 1.000 |
| Total | All reasons | 5 (11.6%) | 3 (6.4%) | 0.615 |
|  | Unknown | 2 (4.7%) | 1 (2.1%) | 0.938 |
|  | No sampling | 1 (2.3%) | 0 (0.0%) | 0.478 |
|  | Sample loss | 1 (2.3%) | 0 (0.0%) | 0.478 |
|  | Sample hemolysis | 1 (2.3%) | 2 (4.3%) | 1.000 |

NA, not applicable.

Note: Values were presented as n (%). P values were determined using chi-square test or Fisher’s exact test, as appropriate.

**Table S5 Demographic characteristics and final diagnoses between patients with and without missing data of anti-Xa activities in the High-dose group (primary analysis population)**

|  | **Patients without missing data of anti-Xa activities**  **(N = 38)** | **Patients with missing data of anti-Xa activities**  **(N = 5)** | ***P* value** |
| --- | --- | --- | --- |
| Demographic characteristics | | | |
| Age (years) | 64.00 (57.00, 68.00) | 61.00 (60.00, 67.00) | 0.879 |
| Age ≥ 75 years | 3 (7.9%) | 0 (0.0%) | 1.000 |
| Female | 11 (28.9%) | 2 (40.0%) | 1.000 |
| Body weight (Kg) | 71.8 ± 13.6 | 68.5 ± 10.7 | 0.611 |
| Body weight < 60 Kg | 9 (23.7%) | 2 (40.0%) | 0.810 |
| Final diagnoses | | | |
| Symptomatic CAD | 8 (21.1%) | 1 (20.0%) | 1.000 |
| Stable angina | 5 (13.2%) | 1 (20.0%) | 0.547 |
| Unstable angina | 3 (7.9%) | 0 (0.0%) | 1.000 |
| Asymptomatic CAD | 19 (50.0%) | 4 (80.0%) | 0.431 |
| Coronary atherosclerosis | 11 (28.9%) | 0 (0.0%) | 0.396 |
| Myocardial bridge | 1 (2.6%) | 0 (0.0%) | 1.000 |

CAD, coronary artery disease; Kg, kilogram.

Note: Values were mean ± standard deviation or median (interquartile range) for continuous data and n (%) for categorical data, as appropriate. P values were determined using two-sample t-test or Mann–Whitney U test for continuous variables and chi-square test or Fisher’s exact test for categorical variables, as appropriate.

**Table S6 Demographic characteristics and final diagnoses between patients with and without missing data of anti-Xa activities in the Standard-dose group (primary analysis population)**

|  | **Patients without missing data of anti-Xa activities**  **(N = 44)** | **Patients with missing data of anti-Xa activities**  **(N = 3)** | ***P* value** |
| --- | --- | --- | --- |
| Demographic characteristics | | | |
| Age (years) | 65.00 (59.50, 71.50) | 63.00 (63.00, 67.00) | 0.948 |
| Age ≥ 75 years | 4 (9.1%) | 0 (0.0%) | 1.000 |
| Female | 14 (31.8%) | 1 (33.3%) | 1.000 |
| Body weight (Kg) | 71.9 ± 12.2 | 71.3 ± 5.0 | 0.937 |
| Body weight < 60 Kg | 5 (11.4%) | 0 (0.0%) | 1.000 |
| Final diagnoses | | | |
| Symptomatic CAD | 6 (13.6%) | 0 (0.0%) | 1.000 |
| Stable angina | 6 (13.6%) | 0 (0.0%) | 1.000 |
| Unstable angina | 0 (0.0%) | 0 (0.0%) | NA |
| Asymptomatic CAD | 27 (61.4%) | 3 (100.0%) | 0.467 |
| Coronary atherosclerosis | 10 (22.7%) | 0 (0.0%) | 1.000 |
| Myocardial bridge | 3 (6.8%) | 0 (0.0%) | 1.000 |

CAD, coronary artery disease; Kg, kilogram; NA, not applicable.

Note: Values were mean ± standard deviation or median (interquartile range) for continuous data and n (%) for categorical data, as appropriate. P values were determined using two-sample t-test or Mann–Whitney U test for continuous variables and chi-square test or Fisher’s exact test for categorical variables, as appropriate.

**Table S7 Anti-Xa activities and rates of target, under-, and over-anticoagulation at different time points after missing data imputation (primary analysis population)**

| **Time-points** | **Treatment** | | ***P* value** |
| --- | --- | --- | --- |
|  | **High-dose**  **(N = 43)** | **Standard-dose**  **(N = 47)** |  |
| 0 min | 0.05 (0.05, 0.07) | 0.06 (0.05, 0.08) | 0.060 |
| < 0.5 IU/ml | 43 (100.0%) | 47 (100.0%) | NA |
| 0.5-1.8 IU/ml | 0 (0.0%) | 0 (0.0%) | NA |
| > 1.8 IU/ml | 0 (0.0%) | 0 (0.0%) | NA |
| 10 min | 1.37 (1.16, 1.50) | 0.94 (0.83, 1.13) | < 0.001 |
| < 0.5 IU/ml | 0 (0.0%) | 1 (2.1%) | 1.000 |
| 0.5-1.8 IU/ml | 43 (100.0%) | 46 (97.9%) | 1.000 |
| > 1.8 IU/ml | 0 (0.0%) | 0 (0.0%) | NA |
| 90 min | 0.79 (0.68, 0.90) | 0.57 (0.50, 0.69) | < 0.001 |
| < 0.5 IU/ml | 0 (0.0%) | 12 (25.5%) | < 0.001 |
| 0.5-1.8 IU/ml | 43 (100.0%) | 35 (74.5%) | < 0.001 |
| > 1.8 IU/ml | 0 (0.0%) | 0 (0.0%) | NA |

IU, international unit; NA, not applicable.

Note: Missing data was imputed through multiple imputation approach (predictive mean matching method) using the mice package in R version 4.5.2, with 50 imputed datasets generated and the modes selected for final imputation. Target, under-, and over-anticoagulation were defined as anti-Xa activities of 0.5-1.8 IU/ml, < 0.5 IU/ml, and > 1.8 IU/ml, respectively. Values were median (interquartile range) for continuous data and n (%) for categorical data, as appropriate. P values were determined using Mann–Whitney U test for continuous variables and chi-square test or Fisher’s exact test for categorical variables, as appropriate.

**Table S8 Anti-Xa activities and rates of target, under-, and over-anticoagulation at different time points (modified intention-to-treat population)**

| **Time-points** | **Treatment** | | ***P* value** |
| --- | --- | --- | --- |
|  | **High-dose**  **(N = 48)** | **Standard-dose**  **(N = 48)** |  |
| 0 min | 0.06 (0.05, 0.08) | 0.06 (0.05, 0.08) | 0.424 |
| < 0.5 IU/ml | 41 (89.1%) | 47 (97.9%) | 0.187 |
| 0.5-1.8 IU/ml | 2 (4.3%) | 1 (2.1%) | 0.970 |
| > 1.8 IU/ml | 3 (6.5%) | 0 (0.0%) | 0.226 |
| 10 min | 1.37 (1.16, 1.53) | 0.94 (0.84, 1.11) | < 0.001 |
| < 0.5 IU/ml | 0 (0.0%) | 1 (2.1%) | 1.000 |
| 0.5-1.8 IU/ml | 46 (100.0%) | 47 (97.9%) | 1.000 |
| > 1.8 IU/ml | 0 (0.0%) | 0 (0.0%) | NA |
| 90 min | 0.79 (0.64, 0.92) | 0.57 (0.49, 0.69) | < 0.001 |
| < 0.5 IU/ml | 0 (0.0%) | 12 (26.7%) | < 0.001 |
| 0.5-1.8 IU/ml | 43 (100.0%) | 33 (73.3%) | < 0.001 |
| > 1.8 IU/ml | 0 (0.0%) | 0 (0.0%) | NA |

IU, international unit; NA, not applicable.

Note: Target, under-, and over-anticoagulation were defined as anti-Xa activities of 0.5-1.8 IU/ml, < 0.5 IU/ml, and > 1.8 IU/ml, respectively. Values were median (interquartile range) for continuous data and n (%) for categorical data, as appropriate. P values were determined using Mann–Whitney U test for continuous variables and chi-square test or Fisher’s exact test for categorical variables, as appropriate.

**Table S9 Anti-Xa activities and rates of target, under-, and over-anticoagulation at different time points (per-protocol population)**

| **Time-points** | **Treatment** | | ***P* value** |
| --- | --- | --- | --- |
|  | **High-dose**  **(N = 37)** | **Standard-dose**  **(N = 44)** |  |
| 0 min | 0.05 (0.05, 0.07) | 0.06 (0.05, 0.07) | 0.109 |
| < 0.5 IU/ml | 37 (100.0%) | 44 (100.0%) | NA |
| 0.5-1.8 IU/ml | 0 (0.0%) | 0 (0.0%) | NA |
| > 1.8 IU/ml | 0 (0.0%) | 0 (0.0%) | NA |
| 10 min | 1.37 (1.16, 1.49) | 0.93 (0.83, 1.04) | < 0.001 |
| < 0.5 IU/ml | 0 (0.0%) | 1 (2.3%) | 1.000 |
| 0.5-1.8 IU/ml | 37 (100.0%) | 43 (97.7%) | 1.000 |
| > 1.8 IU/ml | 0 (0.0%) | 0 (0.0%) | NA |
| 90 min | 0.80 (0.70, 0.91) | 0.57 (0.49, 0.69) | < 0.001 |
| < 0.5 IU/ml | 0 (0.0%) | 12 (27.3%) | < 0.001 |
| 0.5-1.8 IU/ml | 37 (100.0%) | 32 (72.7%) | < 0.001 |
| > 1.8 IU/ml | 0 (0.0%) | 0 (0.0%) | NA |

IU, international unit; NA, not applicable.

Note: Target, under-, and over-anticoagulation were defined as anti-Xa activities of 0.5-1.8 IU/ml, < 0.5 IU/ml, and > 1.8 IU/ml, respectively. Values were median (interquartile range) for continuous data and n (%) for categorical data, as appropriate. P values were determined using Mann–Whitney U test for continuous variables and chi-square test or Fisher’s exact test for categorical variables, as appropriate.
